# Draft Genome Sequence of *Sphingobium chinhatense* Strain IP26^T^, Isolated from a Hexachlorocyclohexane Dumpsite

**DOI:** 10.1128/genomeA.00680-13

**Published:** 2013-08-29

**Authors:** Neha Niharika, Naseer Sangwan, Salar Ahmad, Priya Singh, J. P. Khurana, Rup Lal

**Affiliations:** Molecular Biology Laboratory, Department of Zoology, University of Delhi, Delhi, Indiaa; Interdisciplinary Centre for Plant Genomics & Department of Plant Molecular Biology, University of Delhi South Campus, New Delhi, Indiab

## Abstract

*Sphingobium chinhatense* strain IP26^T^ is a conducive hexachlorocyclohexane (HCH) degrader isolated from a heavily contaminated (450 mg HCH/g soil) HCH dumpsite. IP26^T^ degrades α-, β-, γ-, and δ-HCH, which are highly persistent in the environment. Here we report the draft genome sequence (~5.8 Mbp) of this strain.

## GENOME ANNOUNCEMENT

In order to study the evolution of hexachlorocyclohexane (HCH)-degrading phenotypes at intragenus level among sphingomonads, we have isolated several HCH-degrading and/or -tolerating genotypes, including *Sphingobium chinhatense* strain IP26^T^ from the HCH dumpsite (1) located at Chinhat, Lucknow (26°54′ N and 81°09′ E), India. Gas-liquid chromatography-based HCH isomer degradation analysis (time dependent) revealed that IP26^T^ is a faster degrader of HCH isomers than the prototype bacterium *Sphingobium indicum* strain B90A^T^ (2).

A draft genome sequence of *S. chinhatense* IP26^T^ was obtained by use of Illumina Genome Analyzer IIx and 454 GS FLX titanium platforms, which generated ~1.4 Gb (pair-end) and ~116 Mb (single-end) sequencing data with coverage of 131- and 20-fold, respectively. Raw data were assembled into contigs (*n* = 236, >500 bp) using the ABySS 1.3.3 assembler (3) set at a k-mer size of 61. The assembled genome had an *N*_50_ value of 142 kb and an average GC content of 64.1%. The draft genome was annotated using RAST version 4.0 (4) and NCBI Prokaryotic Genome Annotation Pipeline (PGAP) version 2.1 (http://www.ncbi.nlm.nih.gov/genomes/static/Pipeline.html), which identified 5,703 protein-coding genes and 10 pseudogenes. Eleven rRNA and 66 tRNA genes were also predicted using PGAP annotations. A contig of size 5,392 bp possessing similar coding sequences (CDS) to plasmid pUT2 (5,398 bp) of *S. japonicum* UT26 (5) was also identified. Whole-genome-based average nucleotide identity (ANI) (6) comparisons revealed that *Sphingobium indicum* B90A^T^ (97.98%), *Sphingobium japonicum* UT26S (97.79%), *Sphingobium chlorophenolicum* L-1 (90.55%), and *Sphingomonas* SKA58 (80.29%) are the closest phylogenetic neighbors of IP26^T^.

The potential to metabolize HCH isomers and a wide range of aromatic hydrocarbons is predicted from the genome. For instance, HCH-degrading *lin* genes associated with IS*6100* elements were present in the genome assembly. IS*6100* elements (*n* = 19) were identified using ISFinder (7). A full-length (1 kb) IS*6100* element was observed with *linA*, *linB*, *linC*, and *linR* genes. In addition, a partial (~331 bp) IS*6100* element was also observed with *linF*, *linG*, *linH*, *linI*, and *linJ* genes. The presence of 30 transposases and 24 phage integrases indicates that the genome is subjected to ongoing genetic rearrangement. Furthermore, phenol/toluene and chlorophenol degradation pathways were observed in IP26^T^. Unlike the genome of *S. indicum* B90A^T^ (2), the draft genome assembly of IP26^T^ was found to have a homogentisate degradation pathway. Genes encoding putative metal resistance/efflux proteins, including resistance proteins for lead, mercury, arsenic, copper, cobalt, cadmium, and zinc, were also identified.

Further analysis of this genome, coupled with the metagenomic data from the HCH dumpsite (8, 9), will be used to understand the possible mechanism of acquisition of *lin* genes and other catabolic genes in the sphingomonads, along with the highly diverse microbial community that has been reported from the HCH dumpsite (8, 9). In addition, sequencing and analysis of more HCH-degrading genotypes from the HCH dumpsite will augment the ongoing efforts to understand the pangenomic aspects of this widely distributed genus at the HCH dumpsite and will reveal the ambiguities that exist in the horizontal gene transfer (HGT) of *lin* genes and other catabolic genes involved in the biodegradation of aromatic compounds.

### Nucleotide sequence accession numbers. 

The draft genome sequence of *S. chinhatense* IP26^T^ is available in GenBank database under accession number AUDA00000000. The version described in this paper is the first version, AUDA01000000.
